# Utility of movement disorder society-unified Parkinson's disease rating scale for evaluating effect of subthalamic nucleus deep brain stimulation

**DOI:** 10.3389/fneur.2022.1042033

**Published:** 2023-01-06

**Authors:** Tatsuya Yamamoto, Yoshitaka Yamanaka, Shigeki Hirano, Yoshinori Higuchi, Satoshi Kuwabara

**Affiliations:** ^1^Department of Neurology, Graduate School of Medicine, Chiba University, Chiba, Japan; ^2^Department of Rehabilitation Science, Chiba Prefectural University of Health Sciences, Chiba, Japan; ^3^Urayasu Rehabilitation Education Center, Chiba University Hospital, Chiba, Japan; ^4^Department of Neurological Surgery, Graduate School of Medicine, Chiba University, Chiba, Japan

**Keywords:** Parkinson's disease, deep brain stimulation, movement disorder society-unified Parkinson's disease rating scale, Lin's concordance correlation coefficient, Bland-Altman analysis

## Abstract

**Background:**

The Movement Disorders Society (MDS)-Unified Parkinson's Disease Rating Scale (UPDRS) is increasingly used to assess motor dysfunction before and after subthalamic nucleus deep brain stimulation (STN-DBS).

**Objectives:**

We, therefore, investigated whether the MDS-UPDRS can detect longitudinal changes in motor function after STN-DBS in the same way as UPDRS.

**Methods:**

We examined 21 patients with Parkinson's disease (PD) (mean age 59.2 ± 10.6 years, mean disease duration 12.0 ± 3.0 years) who underwent STN-DBS and whose motor functions were assessed by the UPDRS and MDS-UPDRS before, 3 months after, and 1 year after STN-DBS. We then evaluated the consistency between the scores of Parts II and III of the UPDRS and MDS-UPDRS during the off phase using Lin's concordance coefficient (LCC) and a Bland-Altman plot.

**Results:**

The scores of Parts II and III of both the UPDRS and MDS-UPDRS were significantly decreased 3 months and 1 year after STN-DBS during the off phase. Scores of the UPDRS and MDS-UPDRS showed significant positive correlations before and after STN-DBS. We calculated estimated MDS-UPDRS scores from the UPDRS scores using a regression line and calculated the LCC between the MDS-UPDRS and the estimated MDS-UPDRS scores. The LCC value was 0.59–0.91, which suggests a relatively high consistency between the UPDRS and MDS-UPDRS. The Bland-Altman plot showed that differences between both scores were basically within ±1.96 standard deviations of the difference.

**Conclusion:**

The present preliminary study indicated that the utility of the MDS-UPDRS in evaluating motor function before and after STN-DBS demonstrates its potential equivalency to the UPDRS.

## Introduction

Parkinson's disease (PD) is a neurodegenerative disorder characterized clinically by bradykinesia, rigidity, and resting tremor (1). The occurrence of bradykinesia is necessary for a PD diagnosis. Some patients with PD develop severe motor complications, such as wearing off and dyskinesia, which are difficult to treat with oral anti-Parkinsonian drugs alone. However, motor complications can be dramatically improved by deep brain stimulation (DBS) (2). Since there are several contra-indications for DBS surgery (2), it is crucial to precisely evaluate both motor and non-motor dysfunction, such as cognitive, neuropsychiatric, and autonomic dysfunction, to determine the indications for DBS surgery. Furthermore, detailed post-operative clinical evaluations are also important.

The Unified Parkinson's Disease Rating Scale (UPDRS) has long been used for the clinical evaluation of patients with PD. The revised version of the UPDRS was published by the Movement Disorders Society (MDS-UPDRS) in 2008 (3), while the Japanese version of the MDS-UPDRS was published in 2013.

The MDS-UPDRS is increasingly used to assess motor dysfunction before and after subthalamic nucleus deep brain stimulation (STN-DBS). Chou et al. reported that the MDS-UPDRS tracked motor and non-motor improvement in patients with PD who underwent subthalamic nucleus DBS (STN-DBS) (4). Goetz et al. (5) developed formulas to convert the scores of Parts II and III of the UPDRS to the MDS-UPDRS, which might promote the use of the MDS-UPDRS rather than the UPDRS (5).

However, we do not know whether the concordance between the scores of Parts II and III of the UPDRS and MDS-UPDRS is maintained after DBS surgery.

Although it is an accepted fact that the MDS-UPDRS rating scale is an internationally recognized scale for assessing motor symptoms for PD, and its status might be comparable to that of UPDRS, we do not know whether MDS-UPDRS is the same as the UPDRS scale in examining the changes in the score of ADL (Parts II) and motor symptoms (Parts III) after STN-DBS. It is expected that MDS-UPDRS might detect the changes in the score of ADL (Parts II) and motor symptoms (Parts III) which might be comparable to UPDRS; no studies have ever examined MDS-UPDRS and UPDRS simultaneously before and after STN-DBS. The direct comparison between the score of MDS-UPDRS and UPDRS before and after STN-DBS is necessary to conclude that MDS-UPDRS is the same as the UPDRS scale in examining the changes in the score of ADL (Parts II) and motor symptoms (Parts III) after STN-DBS.

We aimed to clarify whether concordance between the UPDRS scores and the MDS-UPDRS scores of Parts II and III is maintained before and after STN-DBS by examining the UPDRS and MDS-UPDRS simultaneously.

## Method

### Participants

Between January 2014 and November 2020, we prospectively enrolled 21 patients with PD who underwent bilateral STN-DBS at Chiba University Hospital. We evaluated the consistency between the scores of the Japanese versions of Parts II and III of the UPDRS and MDS-UPDRS during the on and off phase using Lin's concordance coefficient (LCC) and a Bland-Altman plot. A PD diagnosis was based on the clinical diagnostic criteria of the United Kingdom PD Society Brain Bank (6). All participants reported medication-resistant fluctuations and complications in motor function. Before enrollment in the study, participants were treated with anti-Parkinsonian medications and with levodopa, decarboxylase inhibitors, dopamine agonists, selegiline, istradefylline, zonisamide, and entacapone. No participants were treated with anticholinergics immediately before or during the study, and motor function in the “on” and “off” phases during treatment was evaluated using the UPDRS and the MDS-UPDRS Parts I, II, III, and IV before, 3 months after, and 1 year after STN-DBS. The same examiner scored each subject at the same time using both the UPDRS and MDS-UPDRS. Pre-operative evaluation of MDS-UPDRS and UPDRS was performed a few months before surgery. The indications for DBS were determined by the medical staff team after pre-operative evaluations were completed. Therefore, the patients did not know whether they had indications for DBS or not during pre-operative examinations. All post-operative assessments were performed under bilateral ON stimulation. We evaluated the concordance between the scores of Parts II and III of the UPDRS and MDS-UPDRS using LCC and a Bland-Altman plot. LCC is a measure of direct concordance of continuous data. LCC is a product of the amount of agreement between two variables and the Pearson correlation coefficient. Therefore, obtaining a high value of LCC requires both good agreement (small difference between two variables) and a strong correlation between two variables. Bland-Altman plots were used to reveal the level of agreement between the scores of Parts II and III of the MDS-UPDRS and the MDS-UPDRS estimated from the UPDRS using regression analysis. Since Parts I and IV are quite different between the UPDRS and MDS-UPDRS, we did not assess the concordance between the scores of Parts I and IV of the UPDRS and MDS-UPDRS. The levodopa equivalent dose (LED) of anti-Parkinsonian medications was calculated according to a previously published method (7).

### Statistical analysis

All data are expressed as means ± standard errors of the mean, and all statistical analyses were performed using SPSS version 28.0 (IBM, Armonk, USA). The magnitude of the clinical responses was evaluated by Cohen's effect size. We performed repeated measures of ANOVA to examine the effect of STN-DBS on the score of UPDRS and MDS-UPDRS 3 months and 1 year after surgery as compared to the pre-operative baseline. A Bonferroni correction was performed for *post-hoc* analysis. The association between the scores of Parts II and III of the UPDRS and MDS-UPDRS was assessed using a regression model. We converted the scores of Parts II and III of the UPDRS into estimated scores of Parts II and III of the MDS-UPDRS according to the regression analysis results using our original data and using the formula developed by Goetz et al. (5). We calculated the LCC which is a product of the amount of agreement between two variables and the Pearson correlational correlation coefficient. The sample size, having 80% power of detecting an effect of STN-DBS on motor functions (Part III of UPDRS) during the off phase before and after surgery, was calculated assuming alpha = 0.05, power = 0.80, mean pre-operative score of UPDRS Part III during the off phase = 55, mean post-operative score of UPDRS Part III during the off phase = 20, and SD = 11. We evaluated the concordance using LCC between the actual scores of Parts II and Parts III of the MDS-UPDRS and estimated MDS-UPDRS derived from the regression line. The minimally acceptable LCC is 0.90. Bland-Altman plot is based on a simple estimation of the mean and standard deviation of differences between two variables. Bland-Altman plots were developed by obtaining the mean of the MDS-UPDRS, estimating MDS-UPDRS derived from the UPDRS score using original data for each subject, and plotting the mean against the difference between the MDS-UPDRS score and the estimated MDS-UPDRS derived from the UPDRS score using our original data. We checked the difference between the MDS-UPDRS and the MDS-UPDRS estimated from the UPDRS regression analysis by paired *t*-tests before performing Bland-Altman plots. After confirming that the difference between the MDS-UPDRS and the MDS-UPDRS estimated from the UPDRS is not statistically significant, we proceeded with Bland-Altman plots. The analysis of LCC and Bland-Altman plots conformed to the methods published by Goetz et al. (5).

### Ethical considerations

The Chiba University Hospital Institutional Review Board approved this study. All 21 participants provided written informed consent, which was obtained during their “on” phase. The ethical standards committee at Chiba University provided approval to implement this study. All participants consented to the use of their examination scores for analysis.

## Results

In all, 21 patients with PD were enrolled in this study (mean age 59.2 ± 10.6 years, mean disease duration 12.0 ± 3.0 years). Of the 21 participants, 12 completed the post-operative clinical evaluation after 3 months, while 11 completed it after 1 year.

The mean LED decreased significantly from the baseline dosage at each follow-up timepoint after surgery (*P* < 0.01). The mean scores of the UPDRS Parts II and III during the off phase and the UPDRS Part IV decreased significantly (*P* < 0.01) at each follow-up timepoint after surgery compared with the baseline score (Table 1). Although the mean scores of the UPDRS Part III during the on phase decreased significantly at each follow-up timepoint after surgery (*P* < 0.01), the scores of Parts I and II of the UPDRS during the on phase did not significantly decrease after surgery (Table 1). Regarding the MDS-UPDRS, the scores of Parts II and III during the off phase and those of Part IV decreased significantly (*P* < 0.01) at each follow-up timepoint after surgery compared with the baseline score (Table 2). The MDS-UPDRS scores of Parts I, II, and III during the on phase did not significantly decrease after surgery. The effect size was large for the scores of Parts II and III during the off phase and for Part IV score in both the UPDRS and MDS-UPDRS (Tables 1, 2). Within a sample size of nine patients, there was an 80% chance of detecting an effect of STN-DBS on motor functions being evaluated by the changes in the score of Part III of UPDRS during the off phase before and after surgery.

**Table 1:** Scores for UPDRS at baseline and follow-up points after surgery (3 months and 1 year after surgery).

**UPDRS**	**Baseline**	**3 months**	**1 year**	***F*-value**	***p*-value** **(pre vs. 3** **months)**	***p*-value** **(pre vs. 1** **year)**	**Cohen's d (pre-3M)**	**Cohen's d** **(pre-1Y)**
Part I	1.93 (0.29)	1.23 (0.26)	1.23 (0.26)	2.11 (*p* = 0.125)	0.233	0.227	0.36	0.37
Part II during off phase	21.52 (0.97)	11.75 (1.637)	13.94 (1.94)	6.18 (*p* = 0.01)	0.039	0.035	1.21	0.88
Part II during on phase	8.59 (0.78)	7.08 (0.90)	8.69 (0.88)	2.55 (*p* = 0.106)	0.508	0.967	0.26	0.02
Part III during off phase	41.14 (1.79)	20.73 (2.18)	23.36 (2.70)	17.57 (*p* < 0.001)	0.005	< 0.001	1.37	1.37
Part III during on phase	18.35 (1.08)	12.49 (1.16)	15.21 (1.33)	2.26 (*p* = 0.130)	0.35	0.603	0.68	0.35
Part IV	7.83 (0.45)	3.58 (0.46)	3.31 (0.46)	31.55 (*p* < 0.001)	< 0.001	< 0.001	1.37	1.5

**Table 2:** Scores for MDS-UPDRS at baseline and follow-up points after surgery (3 months and 1 year after surgery).

**MDS-UPDRS**	**Baseline**	**3 months**	**1 year**	***F*-value**	***p*-value (pre vs. 3 months)**	***p*-value (pre vs. 1 year)**	**Cohen's d (pre-3M)**	**Cohen's d (pre-1Y)**
Part I	8.72 (1.09)	6.23 (1.29)	6.43 (1.04)	1.39 (*p* = 0.25)	0.383	0.424	0.48	0.46
Part II during off phase	25.33 (1.92)	14.43 (2.73)	13.21 (2.22)	6.27 (*p* = 0.011)	0.016	0.007	1.12	1.34
Part II during on phase	8.39 (1.23)	7.92 (1.48)	10.38 (1.83)	0.058 (*p* = 0.944)	0.988	0.88	0.08	0.32
Part III during off phase	48.68 (4.31)	20.93 (4.69)	21.31 (4.13)	14.37 (*p* < 0.001)	0.002	0.002	1.37	1.4
Part III during on phase	19.15 (2.48)	14.00 (2.44)	16.86 (2.91)	1.029 (*p* = 0.38)	0.44	0.918	0.44	0.19
Part IV	10.84 (0.73)	4.08 (0.94)	4.54 (1.21)	15.95 (*p* < 0.001)	< 0.01	0.015	1.88	1.6

We calculated the estimated MDS-UPDRS score from the UPDRS score using a regression line derived from our original data, and then we calculated the LCC of the scores from Parts II and III during the on and off phases between the MDS-UPDRS and the estimated MDS-UPDRS. Although the value of LCC was approximately 0.90, which is the minimally acceptable value (5), some LCC values were below 0.90 (Table 2). We compared the LCC value derived from the regression line using our original data and that from the formula developed by Goetz et al. (5) (Table 3). The formula developed by Goetz et al. (5) is represented in Table 4. For the pre-operative and post-operative scores from Parts II and III during the on and off phase, the LCC value derived from the regression line using our original data was relatively close to that from the formula calibrated for the Hoehn and Yahr (H & Y) stage III developed by Goetz et al. (5), except for the pre-operative scores from Parts II and the post-operative scores from Parts II 1 year after DBS during the off phases.

**Table 3:** Lin's concordance coefficients (LCC) between MDS-UPDRS and estimated MDS-UPDRS calculated from our data and formula developed by Goetz et al. calibrated for Hoehn and Yahr 3 and Hoehn and Yahr 4/5.

**LCC**	**Part II off pre**	**Part II off 3M**	**Part II off 1Y**	**Part II on pre**	**Part II on 3M**	**Part II on 1Y**
Calculated from our data	0.7	0.59	0.93	0.82	0.87	0.94
Calculated from formula developed by Goetz et al. calibrated for Yahr 3	0.63	0.65	0.93	0.83	0.87	0.93
Calculated from formula developed by Goetz et al. calibrated for Yahr 4/5	0.7	0.63	0.85	0.67	0.73	0.83
**LCC**	**Part III off pre**	**Part III off 3M**	**Part III off 1Y**	**Part III on pre**	**Part III on 3M**	**Part III on 1Y**
Calculated from our data	0.76	0.86	0.86	0.88	0.91	0.81
Calculated from formula developed by Goetz et al. calibrated for Yahr 3	0.91	0.85	0.81	0.92	0.89	0.81
Calculated from formula developed by Goetz et al. calibrated for Yahr 4/5	0.91	0.79	0.77	0.83	0.71	0.75

**Table 4:** Conversion formula calculating estimated MDS-UPDRS score from UPDRS score.

	**Pre-operative**	**Three months after surgery**	**One year after surgery**
**MDS-UPDRS Parts II during off phase**			
Our data	(UPDRS Parts II × 1.025) + 4.946	(UPDRS Parts II × 0.646) + 7.299	(UPDRS Parts II × 1.016) + 0.871
The formula calibrated for H & Y stage III (Goetz et al.)	(UPDRS Parts II × 1.0) + 1.5	(UPDRS Parts II × 1.0) + 1.5	(UPDRS Parts II × 1.0) + 1.5
The formula calibrated for H & Y stage IV/V (Goetz et al.)	(UPDRS Parts II × 1.0) + 4.9	(UPDRS Parts II × 1.0) + 4.9	(UPDRS Parts II × 1.0) + 4.9
**MDS-UPDRS Parts II during on phase**			
Our data	(UPDRS Parts II × 0.947) + 1.274	(UPDRS Parts II × 0.908) + 2.046	(UPDRS Parts II × 1.137) + 0.138
The formula calibrated for H & Y stage III (Goetz et al.)	(UPDRS Parts II × 1.0) + 1.5	(UPDRS Parts II × 1.0) + 1.5	(UPDRS Parts II × 1.0) + 1.5
The formula calibrated for H & Y stage IV/V (Goetz et al.)	(UPDRS Parts II × 1.0) + 4.9	(UPDRS Parts II × 1.0) + 4.9	(UPDRS Parts II × 1.0) + 4.9
**MDS-UPDRS Parts III during off phase**			
Our data	(UPDRS Parts III × 1.279) + 1.094	(UPDRS Parts III × 1.024) + 1.142	(UPDRS Parts III × 1.137) + 0.138
The formula calibrated for H & Y stage III (Goetz et al.)	(UPDRS Parts III × 1.2) + 1.0	(UPDRS Parts III × 1.2) + 1.0	(UPDRS Parts III × 1.2) + 1.0
The formula calibrated for H & Y stage IV/V (Goetz et al.)	(UPDRS Parts III × 1.1) + 7.5	(UPDRS Parts III × 1.1) + 7.5	(UPDRS Parts III × 1.1) + 7.5
**MDS-UPDRS Parts III during on phase**			
Our data	(UPDRS Parts III × 1.271) – 0.581	(UPDRS Parts III × 1.352) – 1.061	(UPDRS Parts III × 1.125) + 3.297
The formula calibrated for H & Y stage III (Goetz et al.)	(UPDRS Parts III × 1.2) + 1.0	(UPDRS Parts III × 1.2) + 1.0	(UPDRS Parts III × 1.2) + 1.0
The formula calibrated for H & Y stage IV/V (Goetz et al.)	(UPDRS Parts III × 1.1) + 7.5	(UPDRS Parts III × 1.1) + 7.5	(UPDRS Parts III × 1.1) + 7.5

**Figure 1:**
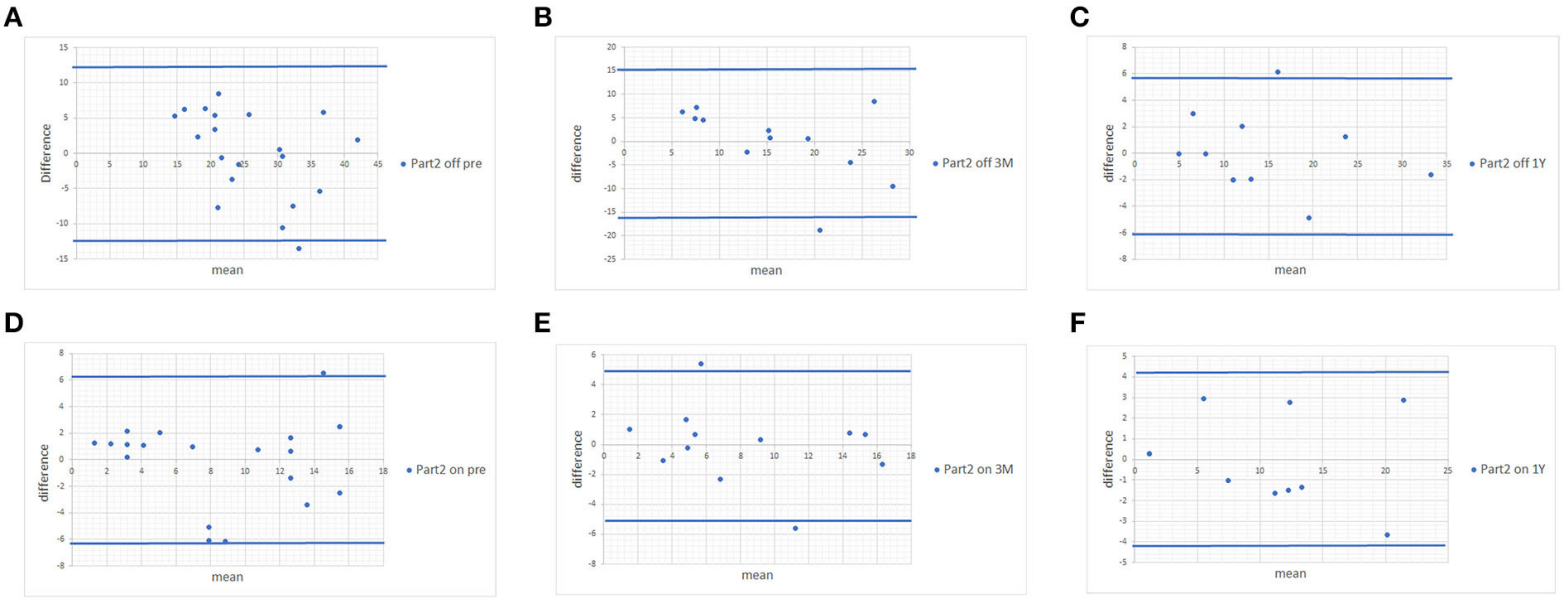
Bland-Altman plots of MDS-UPDRS Part II scores and scores estimated from the UPDRS. **(A)** Pre-operative MDS-UPDRS during the off phase. **(B)** Post-operative MDS-UPDRS 3 months after surgery during the off phase. **(C)** Post-operative MDS-UPDRS 1 year after surgery during the off phase. **(D)** Pre-operative MDS-UPDRS during the on phase. **(E)** Post-operative MDS-UPDRS 3 months after surgery during the on phase. **(F)** Post-operative MDS-UPDRS 1 year after surgery during the on phase. The means of the two scores are displayed on the x-axis, and the difference between the score is displayed on the y-axis. The two blue lines represent the limits of agreement (average difference ± 1.96 standard deviation of the difference).

**Figure 2:**
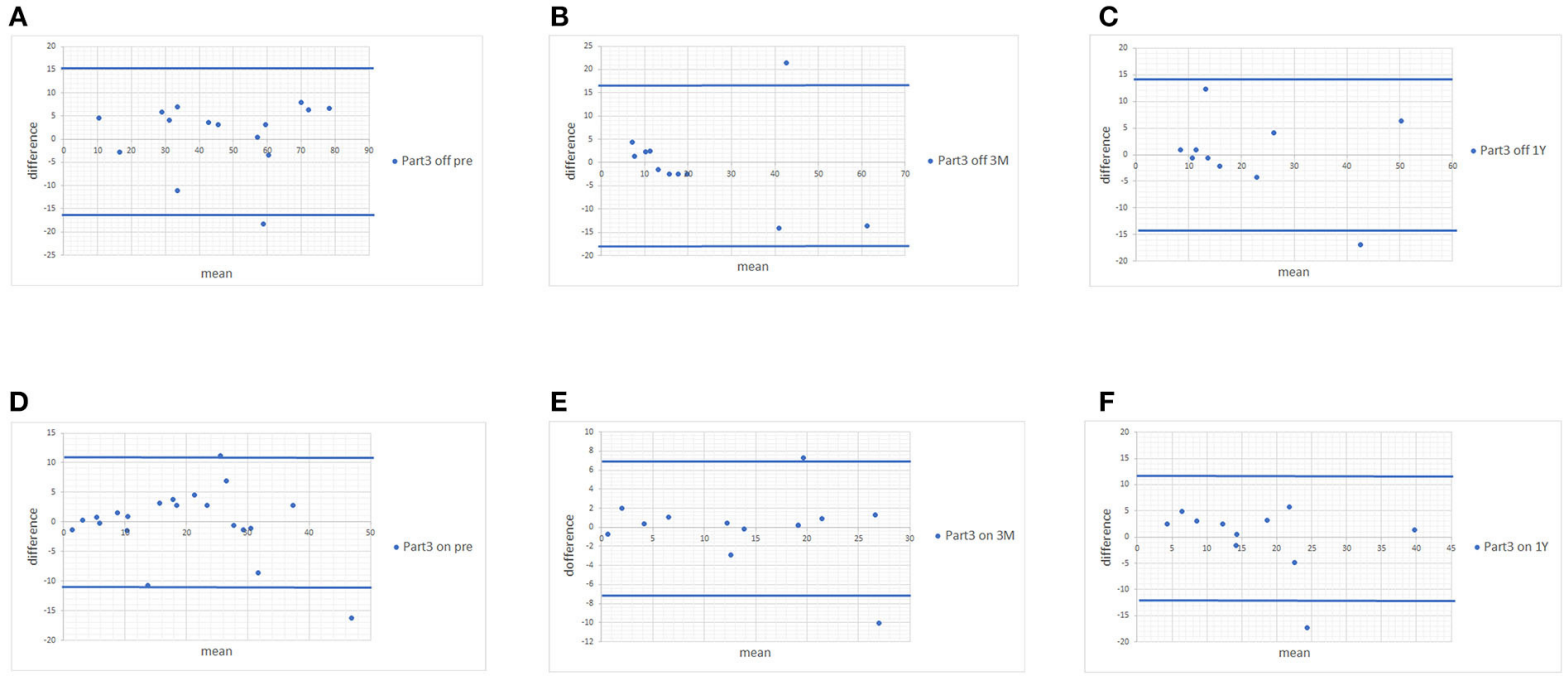
Bland-Altman plots of MDS-UPDRS Part III scores and scores estimated from the UPDRS. **(A)** Pre-operative MDS-UPDRS during the off phase. **(B)** Post-operative MDS-UPDRS 3 months after surgery during the off phase. **(C)** Post-operative MDS-UPDRS 1 year after surgery during the off phase. **(D)** Pre-operative MDS-UPDRS during the on phase. **(E)** Post-operative MDS-UPDRS 3 months after surgery during the on phase. **(F)** Post-operative MDS-UPDRS 1 year after surgery during the on phase. The means of the two scores are displayed on the x-axis, and the difference between the score is displayed on the y-axis. The two blue lines represent the limits of agreement (average difference ± 1.96 standard deviation of the difference).

Since no statistically significant differences were found between MDS-UPDRS and estimated MDS-UPDRS Parts II and III during the on and off phase, we proceeded with Bland-Altman plots. The Bland-Altman plot showed that the differences in both scores were within ±1.96 standard deviations of the difference, which confirmed the strong associations of scores of Parts II (Figure 1) and III (Figure 2) with a few exceptions.

## Discussion

Detailed and appropriate examinations of PD symptoms are essential for determining suitable indications for DBS surgery. In 2008, the revised version of the UPDRS, the MDS-UPDRS, was published and has been increasingly used in the examination of the therapeutic effect of DBS in patients with PD (8). In this study, we aimed to clarify whether concordance between the UPDRS scores and the MDS-UPDRS scores of Parts II and III is maintained before and after STN-DBS by examining the UPDRS and MDS-UPDRS simultaneously.

In this study, we revealed that the UPDRS and MDS-UPDRS showed high concordance, with some exceptions, before and after DBS surgery in patients with PD. The Bland-Altman plot supported the high concordance between the UPDRS and MDS-UPDRS. These results indicated that the MDS-UPDRS might be able to detect the changes in the score of ADL (Parts II) and motor symptoms (Parts III) in the same manner as the original version of the UPDRS. Furthermore, the effect size was large for the scores of Parts II and III during the off phase and the Part IV score in both the UPDRS and MDS-UPDRS, which suggests that the MDS-UPDRS can detect changes in motor scores after DBS surgery.

It should be mentioned that the formula converting the UPDRS to the MDS-UPDRS developed by Goetz et al. (5) depends on the severity, as evaluated by the H & Y stage. In this study, we used a regression line calculated from our original data, since patients with PD in this study exhibited a severe wearing-off phenomenon. As a result, we evaluated the concordance between the UPDRS and MDS-UPDRS scores by the regression line derived from our original data during the on and off phase. We also calculated the LCC value using the formula developed by Goetz et al. (5) and found that the LCC value derived from the regression line using our original data was relatively close to that from the formula calibrated for Hoehn and Yahr (H & Y) stage III developed by Goetz et al. (5), except for the pre-operative scores from Parts II and the post-operative scores from Parts II 1 year after DBS during the off phases. However, the regression equation derived from our original data in this study should be revised with a larger number of patients with PD in a future study.

This study has other limitations, including the small number of participants, some of whom did not complete follow-up evaluations. Due to the small number of participants in this study, this preliminary study should be followed by an examination of a larger number of patients with PD. Some patients at each follow-up timepoint are still currently under investigation. Therefore, a smaller number of patients at each follow-up timepoint compared with the baseline does not indicate a high dropout rate in this study. The small number of participants in this study might have contributed to an exceptionally low LCC value for the Part II scores during the off phase 3 months after surgery. Because simultaneous evaluations of both UPDRS and MDS-UPDRS are time-consuming tasks, pre-operative and post-operative evaluations were performed during hospitalization in our institution. However, because the number of inpatients had to be strictly reduced due to the COVID-19 pandemic in our hospital, it was difficult to perform post-operative evaluations in some patients. Furthermore, it should also be noted that the UPDRS and MDS-UPDRS Part II has not been validated for assessing OFF periods.

Nevertheless, this study indicated that the concordance between the UPDRS and MDS-UPDRS scores might be preserved before and after STN-DBS in patients with PD despite the dramatic decrease in the scores of Parts II and III after surgery. This suggests that the MDS-UPDRS might appropriately detect improvement in motor dysfunction in the same manner as the UPDRS.

## Conclusion

The utility of the MDS-UPDRS in evaluating motor function before and after STN-DBS demonstrates its potential equivalency to the original version of the UPDRS.

## Data availability statement

The raw data supporting the conclusions of this article will be made available by the authors, without undue reservation.

## Ethics statement

The studies involving human participants were reviewed and approved by Chiba University Hospital Institutional Review Board. The patients/participants provided their written informed consent to participate in this study.

## Author contributions

Research project: conception, organization, and execution: TY, YY, SH, and YH. Statistical analysis: design and execution: TY. Review and critique: SH, YH, and SK. Manuscript: writing of the first draft: TY. Review and critique: SH, YH, and SK. All authors contributed to the article and approved the submitted version.
